# A Virtual Screening Platform Identifies Chloroethylagelastatin A as a Potential Ribosomal Inhibitor

**DOI:** 10.3390/biom10101407

**Published:** 2020-10-05

**Authors:** Thomas R. Caulfield, Karen E. Hayes, Yushi Qiu, Mathew Coban, Joon Seok Oh, Amy L. Lane, Takehiko Yoshimitsu, Lori Hazlehurst, John A. Copland, Han W. Tun

**Affiliations:** 1 Department of Cancer Biology, Mayo Clinic, Jacksonville, FL 32224, USA; Qiu.Yushi@mayo.edu (Y.Q.); coban.matt@mayo.edu (M.C.); amy.lane@unf.edu (A.L.L.); copland.john@mayo.edu (J.A.C.); 2 Department of Neuroscience, Mayo Clinic, Jacksonville, FL 32224, USA; 3 Department of Neurosurgery, Mayo Clinic, Jacksonville, FL 32224, USA; 4 Department of Health Sciences Research, Mayo Clinic, Jacksonville, FL 32224, USA; 5 Modulation Therapeutics, Inc., Morgantown, WV 26506, USA; karenh@modulationtherapeutics.com; 6 Department of Chemistry, University of North Florida, Jacksonville, FL 32224, USA; n00902608@ospreys.unf.edu; 7 Division of Pharmaceutical Sciences, Graduate School of Medicine, Dentistry, and Pharmaceutical Sciences, Okayama University, 1-1-1 Tsushima-naka, Kita-ku, Okayama 700-8530, Japan; yoshimit@okayama-u.ac.jp; 8 Department of Pharmaceutical Sciences, West Virginia University, Morgantown, WV 26506, USA; hazlehurst.modulation@gmail.com; 9 Department of Hematology/Oncology, Mayo Clinic, Jacksonville, FL 32224, USA

**Keywords:** chloroethylagelastatin A, agelastatin A, computational screening, ribosome, cancer treatment

## Abstract

Chloroethylagelastatin A (CEAA) is an analogue of agelastatin A (AA), a natural alkaloid derived from a marine sponge. It is under development for therapeutic use against brain tumors as it has excellent central nervous system (CNS) penetration and pre-clinical therapeutic activity against brain tumors. Recently, AA was shown to inhibit protein synthesis by binding to the ribosomal A-site. In this study, we developed a novel virtual screening platform to perform a comprehensive screening of various AA analogues showing that AA analogues with proven therapeutic activity including CEAA have significant ribosomal binding capacity whereas therapeutically inactive analogues show poor ribosomal binding and revealing structural fingerprint features essential for drug-ribosome interactions. In particular, CEAA was found to have greater ribosomal binding capacity than AA. Biological tests showed that CEAA binds the ribosome and contributes to protein synthesis inhibition. Our findings suggest that CEAA may possess ribosomal inhibitor activity and that our virtual screening platform may be a useful tool in discovery and development of novel ribosomal inhibitors.

## 1. Introduction

**Figure 1:**
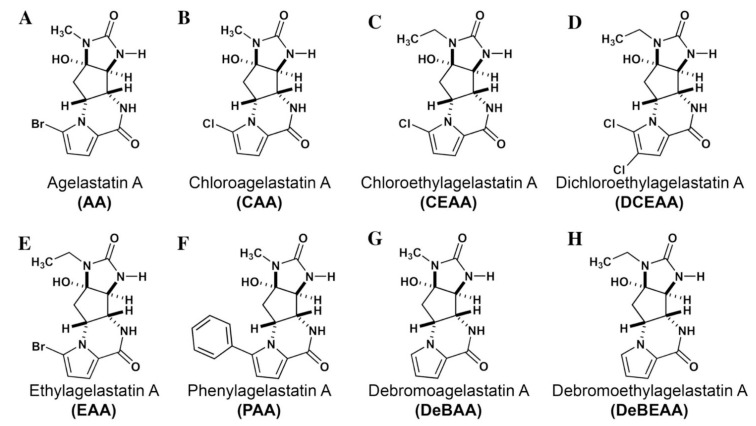
Two-dimensional structures of (-)-Agelastatin A and various analogues analyzed with the docking search algorithm on the ribosome. (**A**) Agelastatin A (AA). (**B**) Chloroagelastatin A (CAA). (**C**) Chloroethylagelastatin A (CEAA). (**D**) Dichloroethylagelastatin A (DCEAA). (**E**) Ethylagelastatin A (EAA). (**F**) Phenylagelastatin A (PAA). (**G**) Debromoagelastatin A (DeBAA). (**H**) Debromoethylagelastatin A (DeBEAA).

Agelastatin A (AA) is a natural compound derived from a marine sponge, Agelas dendromorpha, which has shown significant anti-neoplastic activity [1]. Our pre-clinical central nervous system (CNS) pharmacokinetic and therapeutic evaluation of AA in murine models showed that AA has significant therapeutic activity against CNS lymphoma (CNSL), glioblastoma multiforme (GBM), metastatic breast cancer of brain (MBCB) with a modest CNS penetration (~6.4%) [2]. As there is a dire unmet need for novel therapeutics for brain tumors, we have focused on developing AA analogues with better CNS penetration and therapeutic activity. We have previously designed, synthesized, and evaluated multiple AA analogues [2,3] (Figure 1). 

Based on comprehensive evaluation utilizing in vitro cytotoxicity analysis, in vivo CNS pharmacokinetic analysis, and in vivo analysis in brain tumor models, we have identified three AA analogues (CEAA, CAA, and DCEAA) which possess high CNS penetration (CEAA, CAA, DCEAA- 27%, 16%, and 30.7%; vs. AA- 6.4%) and significant preclinical therapeutic activity against three common brain tumors (CNSL, GBM, and MBCB) comparable to or better than AA (US patent 9464093 B2). CEAA is the lead AA analogue currently under development for therapeutic use against brain tumors. The novel structural modifications of AA associated with improved CNS penetration and therapeutic activity feature one or two chlorine substitutions on the pyrrole moiety (A-ring) at C13 or C14 and ethyl substitution on the D-ring N1-nitrogen atom [3]. 

Mechanism of action of AA and related compounds remained a mystery until recently when it was discovered that AA binds to A-site in ribosome 60S [4]. This seminal discovery represents a major advancement in AA research and opens up opportunities for developing therapeutically active analogues. In this study, we set out to determine whether there is correlation between in silico ribosomal binding capacity of AA analogues and their therapeutic activity. We compared therapeutically active AA analogues (CEAA, CAA, and DCEAA) to therapeutically inactive AA analogues (EAA, PAA, DeBAA, and DeBEAA) by performing a comprehensive virtual screening using layered ribosomal docking technology. The layering consisted of single-precision glide (SP) to extra-precision glide (XP) to Generalized-Born Solvent Accessible (GBSA)/PRIME calculation) [5,6,7,8,9,10,11,12,13,14], which allowed us to generate characterization of structural stability and drug-ribosome interaction fingerprint. From our lead compounds, we developed pharmacophore hypotheses and then performed 3D-quantitative structure activity relationship (3D-QSAR) screening using our larger library of compounds filtering to a candidate pool, which were confirmed with our biological screening and in silico structural elucidations. Biological testing to assess the impact of CEAA on protein synthesis in two lymphoma cell lines was performed for correlation with in silico findings. As presented here, all of these taken together adhere to our virtual and actual screening platform for the generation and optimization of compounds with an *enhanced* clinical profile (ADMET perspective).

## 2. Materials and Methods

### 2.1. Materials for Biological Testing (Cell Culture)

Human B-cell non-Hodgkin lymphoma cell lines, OCI-LY-10 and OCI-LY-3, were cultured with IMDM Modified Medium (GE Hyclone; Logan, UT, USA) supplemented with 20% Fetal Bovine Serum (Life Technologies; Grand Island, NY, USA) and Penicillin/Streptomycin (Corning; Manassas, VA, USA) at 37 °C, in a humidified atmosphere containing 5% CO_2_.

### 2.2. Protein Synthesis Assay

Nascent global protein synthesis was monitored by Protein Synthesis Assay Kit (Caymen Chemicals; Ann Arbor, MI, USA) per manufacturer’s protocol. In short, OCI-LY-10 and OCI-LY-3 were plated in 96-well black plate (Corning, Kennebunk, ME) at 200,000 cells per well and incubated overnight. Cells were pretreated with control (DMSO), CEAA (1 μM), DeBAA (1 μM), AA (1 μM), or Cycloheximide (1 μM) for 1 h; O-Propargyl-puromycin (OPP) was added and incorporated into newly synthesized protein for 2 h. Cells were fixed for 5 min, washed three times, and stained with 5-Fam-Azide solution for 30 min. Nuclei were stained with 4′,6-diamidino-2-phenylindole (Dapi), 1 µg/mL (Thermo Scientific; Rockford, IL, USA). Cells were resuspended in 1 X Tris-buffered saline (TBS) after three additional washes and cells were imaged with BioTek’s Cytation^TM^ 5 Cell Imaging Multi-Mode Reader (ex:490/ex:525) using Gen 5 3.05 software (BioTek; Winooski, VT, USA). Average background (no OPP) RFU was subtracted from RFU of samples (plated in triplicate) and results were normalized to control. 

### 2.3. Statistical Methods for Analysis of Protein Synthesis Data

Statistical significance was determined with Dunnett’s multiple comparisons test by one-way ANOVA Multiple Comparisons using GraphPad Prism 8 software (GraphPad Software; San Diego, CA, USA). Experiments were done in triplicate. Error bars represent SEM; * indicates *p* ≤ 0.05. Either OriginPro 2016 from Origin Lab Corporation or Microsoft Excel 2010 is used for data analysis. The independent-samples t-test was used to test the probability of significant differences between groups. Statistical significance was defined as *p* < 0.05 (*) and statistical high significance was defined as *p* < 0.01 (**). Error bars were given based on calculated SD values where applicable.

### 2.4. Molecular Modeling Methods—Evaluate Shape Fitting Algorithms 

**Figure 2:**
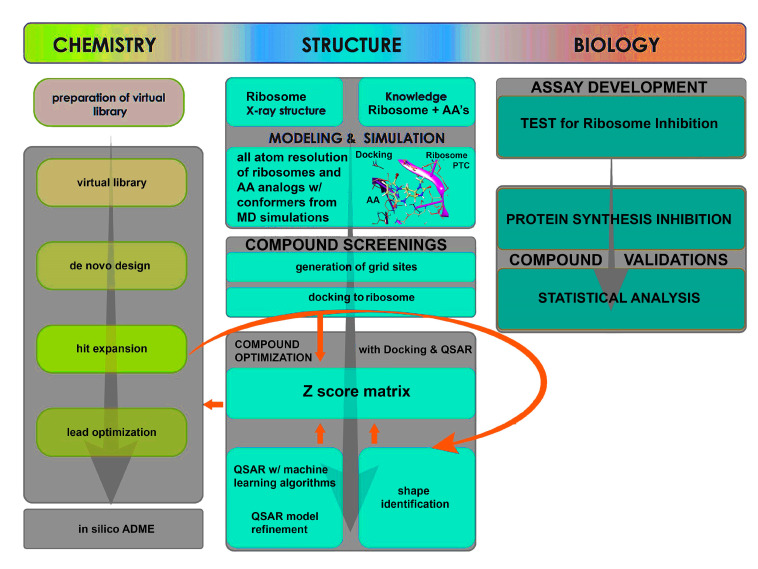
Flow-diagram of virtual screening protocol for ribosome interrogation with AA and AA analogs. Chemistry to Structure studies to Biological validation scheme is shown. The left-most column contains all the chemistry protocols for preparing the AA compound library for virtual screening in a structure-based drug discovery approach that relies on docking. The central column describes the various components that are required for docking the compounds with the X-ray structure for the ribosome and how it is scored for validation with the Biological testing. The right-most column indicates all of the biological assays and screening completed for assessment of AA and AA analogs effect on ribosome inhibition within cells.

Shape similarity models were generated for each compound versus the known inhibitors of AA or AA analogues, such that upon superposition of the known compound (A) and the de novo compound (B), we arrive at the following measurement for jointly occupied volume, $V_{A\cap B}$, which is normalized by the total volume, $V_{A\cup B}$, thus giving the normalized shape similarity $Sim_{AB}$ ranging from 0 to 1. Thus, $Sim_{AB}=V_{A\cap B}/V_{A\cup B}$, where A is the known inhibitor AA or (CEAA, CAA, DCEAA, and so forth) and B is the unknown, de novo compound, were computed for 1000 s of generated poses. Finally, a pairwise method was employed for faster calculations (600 conformers per second) [15,16,17,18,19]. Each compound is allowed to generate 500 conformers, retaining 20 conformers per rotatable bond, allowing the amide bonds to vary conformation, to maximize shape-matching likelihood. We also computed volume for both pharmacophore types and atom types using the Macromodel definition for atom typing [20]. When assembling our data into a database, we wrote out 4 alignments per ligand, filtering out conformers with similarity below 0.7 and then compiled all the shape data together selecting the top shape matching compounds for retention and excluding all lower scoring hits. Various models for chemical shape can also be employed to screen both shape and chemical information simultaneously [15,16,17,18,19] (Figure 2, center column).

### 2.5. Evaluate De Novo Ligand Generation for AA Analogues

**Figure 3:**
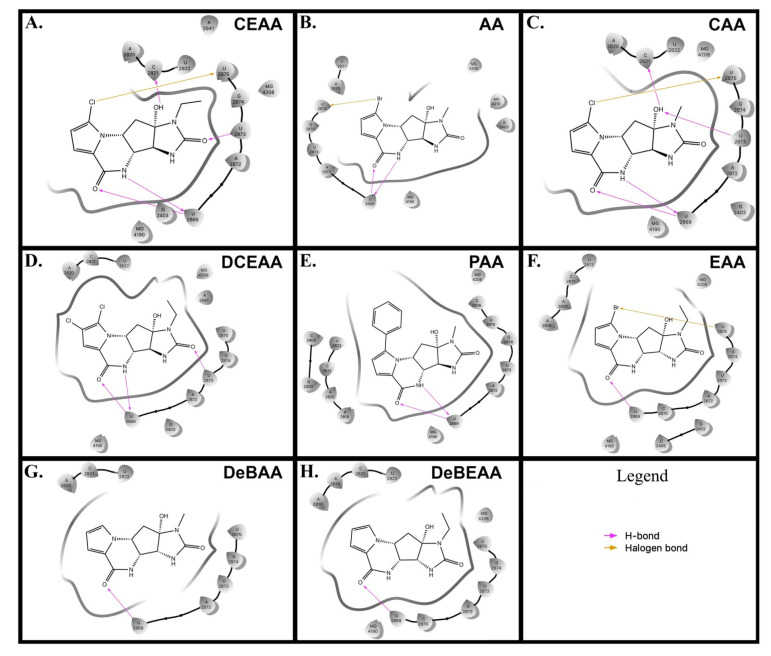
Two-dimensional ligand interaction diagram for peptidyl transferase center (PTC) of the ribosome with AA analogs. Comprehensive list for all 2D Ligand Interaction Maps for CEAA, AA, CAA, DCEAA, PAA, EAA, DeBAA, and DeBEAA with the ribosome PTC is given. Arrows indicate key interactions or other colormetric cues as indicated in the legend on bottom right. **(A)** CEAA is shown with extra binding features that may enhance its activity. **(B)** AA is shown bound to the PTC with the indicated interactions. **(C)** CAA is given for its PTC binding. **(D)** DCEAA has some interesting repositioning due to its extra chloro- moiety. **(E)** PAA has a frustrated orientation due to the large phenyl moiety. **(F)** EAA reveals loss of interactions. **(G)** DeBAA has a very weak hold in pocket with only minor contact. **(H)** Similarly, DeBEAA is weakened in the binding pocket.

**Figure 4:**
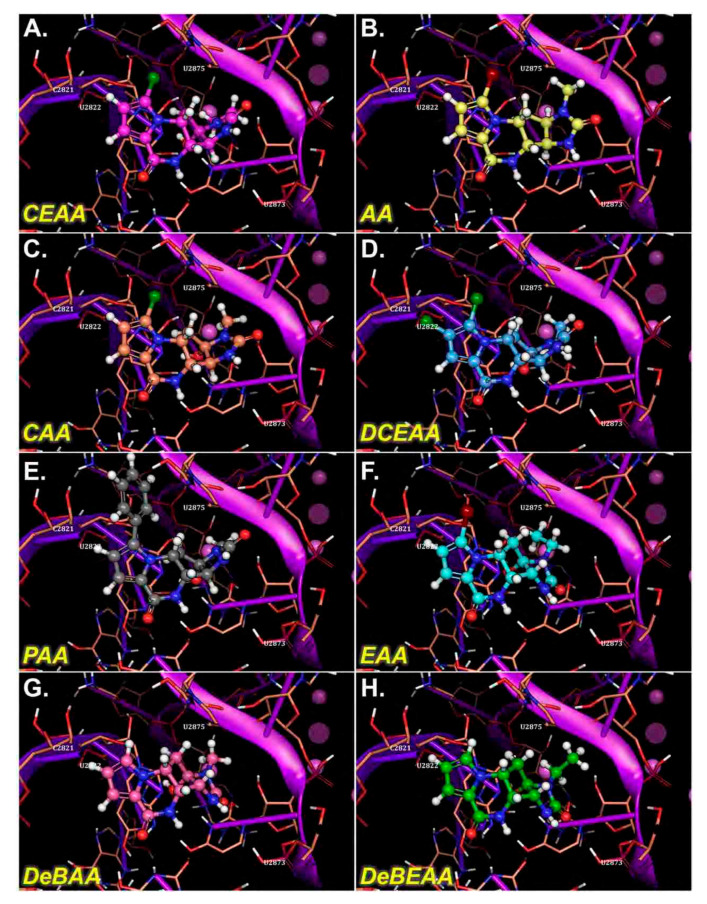
Three-dimensional docked images for AA, CAA, CEAA, EAA, PAA, DCEAA, DeBAA, and DeBEAA with ribosome’s PTC region (prior to Molecular Mechanics-Generalized-Born Solvent Accessible (MM-GBSA) energy calculations). Docking assigns the compounds pose or position in the PTC. The potent derivatives, CEAA, AA, DCEAA and CAA have a similar orientation in their binding and other less potent derivatives lack matching overlap. As the docking is machine driven to the optimal score possible and alternative positions drop with increasingly positive scores, these orientations illustrate the effect subtle changes have on compound efficacy and orientation. Here, the relevant interactions between ribosome and the AA that form important binding sites are shown. (**A**) AA is shown bound in the ribosomal PTC, which has good docking pose that matches the X-ray structure (see two-dimensional ligand interaction map for details on binding residues). (**B**) CEAA shows the critical residues needed for binding to PTC. (**C**) DCEAA is shown with the PTC region surrounding. (**D**) CAA has some similar binding characteristics to that of AA on the PTC. (**E**) PAA is given as bound in the ribosome. (**F**) EAA has weak binding in the PTC. (**G**) DeBAA is shown docked into PTC. (**H**) DeBEAA has weak binding at the PTC.

We created a pool of novel compounds by first examining all the AA analogues with known activity, which includes CEAA, CAA, DCEAA and the other AA analogues (Figure 1, Figure 3 and Figure 4). Using our methodology of combining multiple data sources for an accelerated drug discovery process, we generated multiple new compounds using scaffold manipulation. First, we separated the cores (“core separation”) from all external moieties, leaving just the central core features. Then, using our Library Generator, we combined potential fragments from varied sources (core libraries, in-house fragment libraries, and de novo scaffold manipulations), which are then inserted back into the core slots (Core_1_, Core_2_, or Core_3_) exhaustively. Each new core is probed with alternative chemistry on the edges and placed into the appropriate similarity-based pool (pool_1_, pool_2_, or pool_3_). Each pool gets chemical filtration via energy minimization, correct bond orders, and ligand preparation with LigPrep. These pools are then combined into a central de novo ligand pool. The entire pool of ligands is expanded to allow for generation of tautomers where appropriate, ionization states over a valid range of pH values, and isomerizations. Any reactive functional groups are screened and removed from the dataset. At this point, Z-scoring filtering is applied as a reductive filter for AA-ligand-like specificity.

### 2.6. Molecular Dynamics Simulations

Molecular dynamics (MD) was completed on each compound with a region around it for conformational sampling; the primary purpose of MD, in this scenario, is examining any conformational variability that may occur. Briefly, each system was minimized with relaxed restraints, using either steepest descent (SD) or PRCG, and equilibrated in solvent with physiological salt conditions, as shown in the literature [13,21,22,23]. The protocol for refinement included the following steps: (1) Minimization with explicit water molecules and ions, (2) Energy minimization of the entire system, and (3) MDS for >10 ns to relax to the force field (OPLS3/Amber) [24,25]. Following the refinement protocol, production simulations were completed to collect data, an additional MD production length completed; this overall production, which was completed to allow the molecules to relax within the system region.

OPLS3 (Desmond)/Amber (NAMD2) force fields were used with the current release of Nanoscale Molecular Dynamics 2 engine [25,26]. The simulated system, including hydrogens, consisted of solvation using SPC/E water and ions. In all cases, we added physiological salt conditions with a solvent of 150 mM Na^+^ Cl^−^. SPC/E water molecules were added around the system at a depth of 15–18 Å from the edge [27]. Our protocol has been previously described in the literature [13]. Simulations were carried out using the particle mesh Ewald technique with repeating boundary conditions with a 9 Å nonbonded cut-off, using SHAKE with a 2 fs timestep. 

Pre-equilibration was started with three stages of minimization with 10,000 steps of SD, PRCG, relaxing restraints, then followed by 1000 ps of heating under MD, with the atomic positions of nucleic and protein fixed. Then, two cycles of minimization (5000 steps each) and heating (1000 ps) were carried out with soft restraints of 10 and 5 kcal/(mol·Å^2^) applied to all backbone atoms and metals. Next, 5000 steps of minimization were performed with solute restraints reduced to 1 kcal/(mol·Å^2^). Following that, 400 ps of MDS were completed using relaxing restraints (1 kcal/(mol·Å^2^)) until all atoms are unrestrained, while the system was slowly heated from 1 to 310 K using velocity rescaling upon reaching the desired 310K during this equilibration phase. Additionally, NPT equilibration was established using velocity rescaling for >10 ns. Finally, production runs of MD were carried out with constant pressure boundary conditions (relaxation time of 1.0 ps) for over 500 nanoseconds. A constant temperature of 310 K was maintained using the Berendsen weak-coupling algorithm with a time constant of 1.0 ps. SHAKE constraints were applied to all hydrogens to eliminate X-H vibrations, which yielded a longer simulation time step (2 fs). Our methods for equilibration and production run protocols are in the literature [12,22,28,29]. Translational and rotational center-of-mass motions were initially removed. Periodically, simulations were interrupted to have the center-of-mass removed again by a subtraction of velocities to account for the “flying ice-cube” effect [30]. Following the simulation, the individual frames were superposed back to the origin, to remove rotation and translation effects. 

## 3. Results

### 3.1. Virtual Screening of AA and AA Analogues on Ribosomal A-Site Peptidyl Transferase Center 

**Figure 5:**
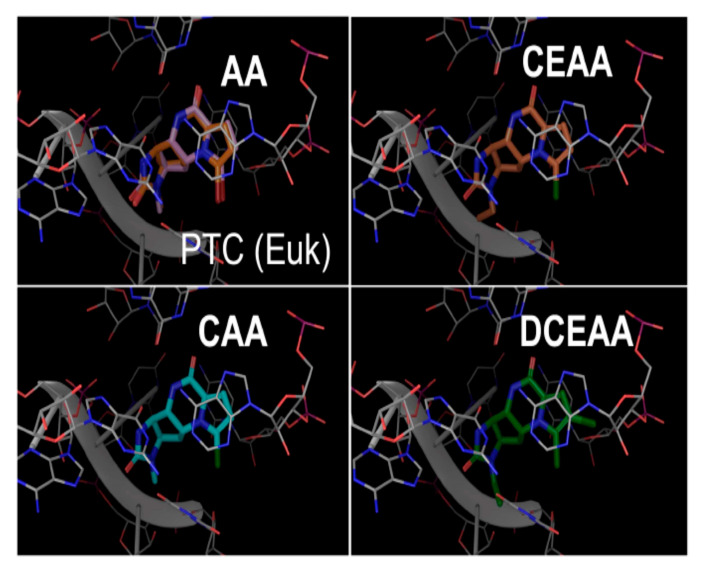
Euk ribosome bound by AA analogues. Top Left: Side-by-side comparison of recently published AA crystal structure (PDB code: 5MEI) (Orange) and our AA docked (Pink) in Euk ribosome, Bottom Left: Docking position of CAA in Euk ribosome (Cyan), Top Right: CEAA (Orange) is the strongest binder with Euk ribosome, Bottom Right: DCEAA (Green) and PAA (not shown) for negative control docked in Euk ribosome. A complete depiction for all AA analogs docked with ribosome PTC is given in Figure 4.

Recent publication indicated that AA is a ribosomal inhibitor, which binds to the A-site [4]. Our docked compounds most likely blocked tRNA binding into the A-site [31,32,33,34,35,36,37,38,39,40,41,42,43,44,45]. From these screens, we examined the following regions: (1) Prokaryotic PTC) from large subunit (50S *E. coli*); (2) Eukaryotic mammalian PTC from large subunit (60S human) (Figure 5). After confirmation of PTC as the ultimate ribosomal binding site by AA, we proceeded to virtual screening of seven AA analogues (Figure 1). 

We compared docking poses of the AA analogues as docked in the Eukaryotic (Euk) ribosome with grid site built upon the existing X-ray structure for the recent AA-ribosome co-crystal [4] and found all of our ligands (including AA) bind in common pharmacophore shown for AA from the X-ray structure (Figure 4). CEAA through DCEAA (Figure 4A–D) have very similar orientation in three-dimensional position respective to the ribosome. However, as we progress to DeBAA and DeBEAA, we find the inhibitors dither more with respect to three-dimensional positioning as compared to AA (Figure 4G–H). Moreover, when we further examine the Euk ribosome with comparison to the published position for AA from the X-ray result (rather than docked) [4], we find our top performers (CEAA, CAA, DCEAA) all possess common respective orientation to that observed within the X-ray positioning for AA (Figure 5). This result comes following our detailed energetics assessment that allows for minor adjustments (post docking movements) to achieve the most likely ideal *pose*. This pose shows good accordance with the experimental findings [4] (Figure 5), and helps us map out critical ligand-enzyme interaction maps in two-dimensions (Figure 3). Further details of this are in sections below.

### 3.2. Ribosomal Docking Reveals that Therapeutically Active AA Analogues Possess Strong Ribosomal Binding Capacity

We performed an analysis of AA and seven AA analogues (Figure 1) to search for interactions with ribosome (bacterial and eukaryotic). Out of seven AA analogues, CEAA, DCEAA, and CAA were previously shown to be therapeutically active whereas EAA, PAA, DeBAA, and DeBEAA were therapeutically inactive [2,3,46]. To undertake this large screen, we utilized a brute force method (layered docking from SP to XP), evaluation with Generalized-Born Solvent-Accessible/Poisson-Boltzmann (GBSA/PB) calculations, and complete three-dimensional analyses for interactions using physics-based computational calculations to generate 2D-ligand interaction maps (interaction fingerprint) (Figure 3; Figure 5).

We performed docking using “building up—filtering down” technique iteratively for AA and seven AA analogues and a decoy. Our incremental build-up of the level of accuracy, from SP to XP and GBSA/prime energy calculations has been previously reported [5,8,10,11,12,47,48,49,50,51]. We docked the AA analogues with the ribosome at the same site as the AA-ribosome X-ray structure to see if similar binding patterns emerged as was shown for AA-ribosome X-ray structure [4], which has been shown to inhibit the ribosome [4] (Figure 3 and Figure 5). Based on the similarity in the 3-dimensional docked binding pose, it would not be unexpected that the mode-of-action is nearly identical. 

Following the rigorous docking protocols, we subjected the systems to high quality assessments using Prime energy calculations and ranked using additional Prime calculations [52,53] (Figure 3 and Figure 4, Table 1). We deduced 2D interaction fingerprints for all analogues (Figure 3). The results from GBSA/prime give us the basis for determining the favorable energies of interaction with ribosome, which is indicative of ribosomal binding affinity. AA and seven AA analogues, based on ribosomal binding affinity, can be ranked from good to poor are as follows: CEAA, AA, DCEAA, CAA, PAA, EAA, DeBAA, and DeBEAA (Table 1). Therapeutically active analogues (CEAA, DCEAA, and CAA) are found to have strong ribosomal binding whereas therapeutically inactive analogues (EAA, PAA, DeBAA, and DeBEAA) do not bind ribosome well. CEAA has the highest ribosome binding affinity, even higher than AA. For comparative analysis, we also performed CSM affinity ligand-protein binding calculations and report the Glide-XP docking scores (Table 1). An alternative conformation for the poorer ligands was also reported and minor pose differences in the top four ligands, which were discarded, but indicated flipping of the poorer binding compounds due to a lack of sufficiently strong interactions. Overall, the data reflected in the table show a best to worst ranking as CEAA (best), AA, CAA, DCEAA, PAA, EAA, DeBAA, and DeBEAA (worst). The bottom three, which includes EAA, DeBAA and DeBEAA are similarly poor when looking at GBSA results. CEAA has strongest performance overall (Table 1, Figure 3 and Figure 4).

### 3.3. Structural Basis for Drug-Ribosome Interactions (3D-QSAR) Identifies Key Fingerprint Features (2D-Ligand Interaction Maps)

**Figure 6:**
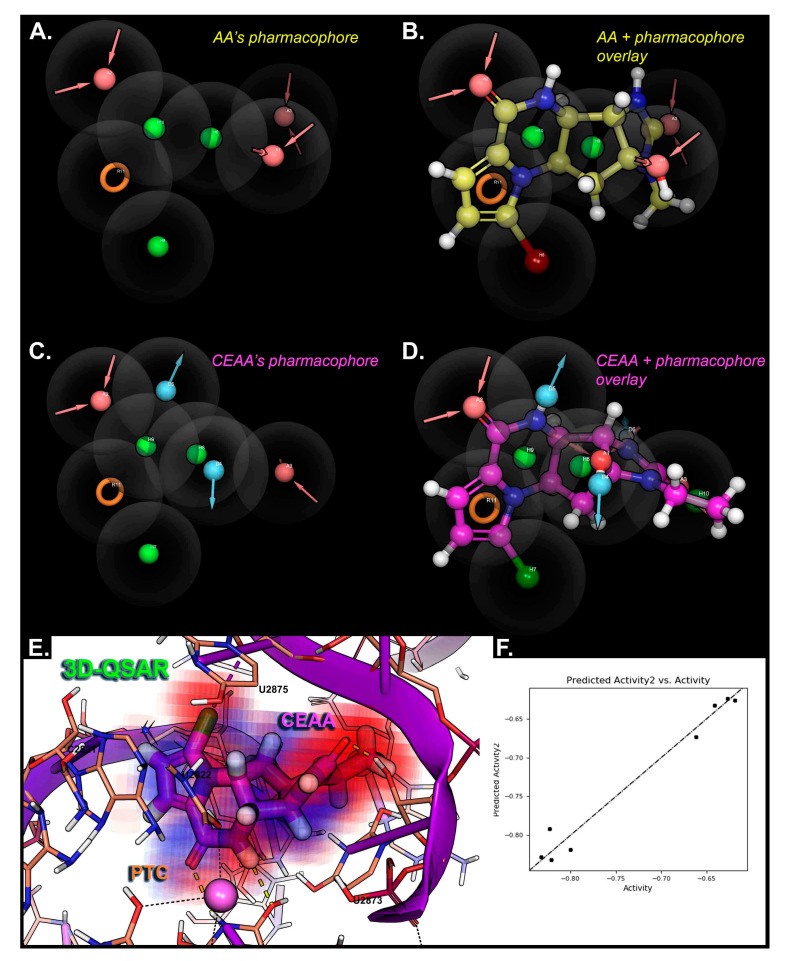
Three-dimensional-quantitative structure activity relationship (QSAR) modeling for AA analogues binding within the PTC of the ribosome. (**A**) AA pharmacophore hypothesis is generated for the most useful features that can selectively bind and be used for 3D-QSAR modeling. (**B**) Overlay of the AA compound structure with the pharmacophore features for AA that have some interactional basis with PTC. (**C**) CEAA pharmacophore hypothesis is given for features most commonly occurring in PTC binding during modeling and dynamics studies. (**D**) Overlay of the CEAA compound structure with the pharmacophore features having an interactional basis with PTC. (**E**) 3D-QSAR map for CEAA within the PTC as bound. Here, the factors from a PLS-2 quantify the distribution of contributions: electrostatics (electron-withdrawing), hydrophobic/non-polar, and H-bond donors. The combined effects are “voxelized” into 0.5-Å^3^ units with red-to-blue color scheme giving the essential interaction sites on CEAA with the PTC (ribosome). (**F**) Graph for correlation between the prediction and actual activities of the 8 AA analogs is given, where R^2^ > 0.97, SD 0.019, RMSE 0.01, and Pearson 0.987. This demonstrates the accuracy of our compound affinity prediction in this particular system.

Modeling AA and AA analogues using a statistical mechanics approach and molecular mechanics with simulations gives improved results over a docking only approach [54]. Three dimensional-QSAR modeling was completed using pharmacophore maps for lead compounds with the PTC from the ribosome (Figure 6), which is described in more detail in the supplemental materials. The outcome of the 3D-QSAR maps regions of importance in 3D-space for designing and optimizing lead compounds, where CEAA has an improvement over AA that comes from the extra features that are PTC binding (Figure 6C–E) (added donors and change in halogen feature).

Additionally, small simulations were completed to study affinity of the compounds for ribosomal nucleic acid (Figure 4) as we previously published [20,21,46]. In the case of simulation, the water box size consists of ~2 million atoms; giving each system 18 Å-to-edge of box. Each system was bound with the compounds or a vacant enzyme. The particle is free to tumble and migrate during simulation; however, periodic boundary conditions with Particle-Mesh Ewald are utilized to ensure accuracy. Our techniques have been described [10,12,13,23,50,55,56,57] (see Methods 2.8). When examining the drug-ribosome interactions up close (Figure 3 and Figure 4), the captured drug has multiple interactions via Van der Waal (VdW) contacts with the ribosome nucleotides, but additional contacts specific to CEAA over that of AA enhanced the binding. We found that the CEAA affinity compared to AA increases by 47% to over 121% through the course of various NPT simulations. CEAA is preferred to AA with the ribosomal binding partner, but the degree of preference varies (Table 1, Figure 3 and Figure 4). Simulation details for the entire complex are provided in supplemental materials. Free energy (∆G_association_) of binding were implemented within the software algorithm considering factors, such as: lipophilicity, displacement of water, hydrogen bonding and electrostatic interactions, and metal ion/ligand interactions as favorable interactions, while the desolvation of polar or charged groups, restriction of motion, and the entropic cost of binding adversely affect free energy [54,58,59,60] (Table 1). These energetic measurements help us determine its combined free energy associated over time.

### 3.4. Modeling Shows Structural Underpinnings for Drug-Ribosome Interaction for AA Analogues 

Detailed interactions of AA versus CEAA show that CEAA exhibits an additional hydrogen bonding between carbonyl oxygen 2-position on the imidazolone D ring and the nucleobase and N-H of nucleotide U2873 on the ribosome (Figure 3A,C). Both AA and CEAA have halogen atom interactions (Br and Cl, respectively) with U2875 at the pi-cloud electrons from the uracil. Likewise, the -OH at 5-position on AA and CEAA have interactions with the same U2873 oxygens but mitigates via the hydrogen on the -OH to pair via hydrogen bonding. Both AA and CEAA have the same -NH atoms and carbonyl oxygen (positions 9 and 11, respectively) that interact at U2869 nucleobase, which occurs with the AA/CEAA 10-position -NH and uracil base carbonyl oxygen and the AA/CEAA 11-position carbonyl oxygen and the uracil base nitrogen (Figure 3). 

Overall, the extra bond for CEAA offers greater stability and enhancement for the drug-ribosome interaction, which would explain the calculated improvements and the observed biological findings. Likewise, the detailed observations for DCEAA, CAA, and PAA are demonstrative of a weaker set of interactions (Figure 3). CAA has the closest resemblance to AA with the same observed interactions at the 2-, 9-, and 11-position CAA atoms but the halogen is not optimally positioned for a strong and long-lasting pairing, while it is substituted with a switch to the 5-position -OH with C2821 via displacement, pi-stacking, and steric interactions (Figure 3). However, this is less potent than the CEAA interactions with ribosome. DCEAA and PAA are found to maximize steric interactions and buried from solvent similar to the AA or CEAA analogs, but lose much of the exact interactions. These interactions are likely still present transiently, but the strongest pairs observed for these two compounds are the 10-position carbonyl oxygen in the B-ring with U2869 for PAA and 3-position -NH on the D-ring with U2869. Though DCEAA does not show so many strong maintained bonds as AA and CEAA for all of the poses from docking, it does maintain a strong consistent shell of nucleotides to bury the compound, which includes G2403, A2820, A2821, U2869, C2870, A2872, U2873, G2874, and U2875 that surrounds it tightly. In addition, the 3-position -NH from DCEAA has similar U2869 interactions as found in AA and CEAA.

Therapeutically inactive AA analogues (EAA, PAA, DeBAA, and DeBEAA) have fewer organized nucleotides around the compounds, less buried surface, and fewer direct interactions (H-bonds, VdW, Pi-cloud, etc.). Thus, these compounds are less optimally bound and are predicted to more readily dissociate from the ribosome than bind. EAA some adjacent nucleotides with a halogen bond via the Bromine and U2875 and the prototypical interaction between U2869 and the carbonyl oxygen (positions 11) from EAA, while DeBAA and DeBEAA only maintain one nucleotide consistently present in all orientations (U2869 nucleobase nitrogen ) that bind via hydrogen bonding at the 11-position carbonyl oxygen. Lastly, PAA is the nearly the same as DeBAA with binding via U2869 nucleobase nitrogen and the 11-position carbonyl oxygen (hydrogen bonding), as well as the hydrogen from the 10-position -NH of PAA with the backbone oxygen from U2869 (Figure 3 and Figure 4). PAA demonstrates mixed computational results and is suspected mechanism of inactive experimental results is due to the bulky hydrophobic appendage (-phenyl) on the AA ring structure.

### 3.5. CEAA is an Inhibitor of Protein Synthesis 

**Figure 7:**
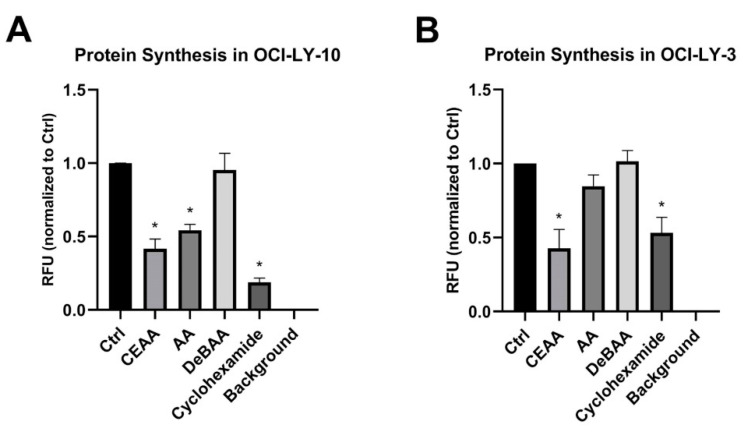
Chloro-ethyl agelastatin A (CEAA) significantly inhibits protein synthesis in two diffuse large B-cell lymphoma cell lines. (**A**) Protein synthesis, incorporation of O-propargyl-puromycin (OPP), was reduced with CEAA (1 µM) and Agelastatin A (AA, 1 µM) treatment of OCI-LY-10 cells compared to DMSO (Ctrl). A similar inhibition was not observed with an in-active AA analogue (DeBAA, 1 µM). Cyclohexamide (1 µM), a known translation inhibitor, was used as a positive control. (**B**) Similar results were observed in OCI-LY-3 cells, but AA treatment did not effectively reduce protein translation. Experiments were done in triplicate. Error bars represent SEM; * indicates *p* ≤ 0.05.

To determine if the increase predicted binding of CEAA to the ribosome correlated to a decrease in protein synthesis relative to AA we examine global protein synthesis. Human B-cell non-Hodgkin lymphoma cell lines, OCI-LY-10 and OCI-LY-3, were pre-treated with Control (Ctrl), CEAA, AA, or inactive DeBAA then incubated with O-Propargyl-Puromycin (OPP), a reagent that incorporates into C terminus of new polypeptides generated during translation. CEAA reduced protein synthesis compared to Ctrl in OCI-LY-10 and OCI-LY-3 by 58% and 57%, respectively. As forecasted by molecular modeling of drug/ribosome interaction, CEAA was more active than AA. Nascent proteins levels were only reduced by 46% to 15% in OCI-LY-10 and OCI-LY-3 cell lines, respectively, with AA treatment; in-active DeBAA did not significantly affect protein translation. Cycloheximide, a known inhibitor of translation, was used as a positive control (Figure 7). 

Collectively, these results suggest that CEAA functions in a similar manner as AA by inhibiting ribosomes and ultimately protein synthesis in two human lymphoma cell lines. In vitro global nascent protein analysis demonstrated that CEAA was more efficacious than AA while DeBAA was inactive confirming the predictive results of our molecular modeling. 

## 4. Discussion

### 4.1. Ribosomal Binding Capacity of CEAA Correlates with Inhibition of Protein Synthesis

In this study, we performed virtual screening and ribosomal docking to determine the correlation between the ribosomal binding affinity of AA analogues and their therapeutic activity. We showed that therapeutically active analogues (CEAA, DCEAA, and CAA) have strong ribosomal binding affinity whereas therapeutically inactive analogues (EAA, PAA, DeBAA, and DeBEAA) have poor affinity for ribosomal binding. CEAA has the best ribosomal binding affinity even exceeding AA. We went on to show by biological testing that CEAA inhibited protein synthesis. This data suggests that ribosomal inhibition is a mechanism of action for therapeutic activity of CEAA [4]. 

### 4.2. Structural Modifications of AA 

The novel structural modifications in therapeutically active AA analogues (CEAA, DCEAA, and CAA) feature one or two chlorine substitutions on the pyrrole moiety (A-ring) at C13 or C14 with or without ethyl substitution on the D-ring N1-nitrogen atom [3]. Based on the crystal structure of AA-ribosome complex [4], it has been shown that chlorine substitution on C13, as in CEAA and CAA, promotes stronger interactions with ribosome. Chlorine substitutions on both C13 and C14, as in DCEAA, preserve ribosomal binding affinity. Ethyl substitution on N1 atom, as in CEAA and DCEAA, does not have any impact on ribosome binding. It is worth noting that structural modifications in therapeutically active AA analogues also result in significantly improved CNS penetration as previously shown [2,3]. We also revealed that therapeutically inactive AA analogues (EAA, PAA, DeBAA, and DeBEAA) have structural modifications that lead to loss of critical interactions required for stable binding to the ribosome resulting in loss of therapeutic activity. This insight can be used to develop therapeutic agents with high CNS penetration and ribosomal targeting.

## 5. Conclusions

Our platform for virtual screening and ribosomal docking can be applied for discovery and development of novel ribosomal inhibitors (Figure 2), which we have previous explored for understanding ribosomal mechanisms [61]. It is highly scalable as it can accommodate screening of large sets of compounds followed by selection and ranking of best candidates based on the GBSA/PB results. The platform is versatile as it can analyze and predict ribosomal binding affinity of candidate compounds and generate structural fingerprints to enable development of novel analogues. Using this technique, we are currently generating more novel AA analogues. Thus, we showed that ribosomal binding is mechanistically important for anti-neoplastic activity of AA analogues and that CEAA is a novel ribosomal inhibitor, more potent than AA. We plan to improve the platform by incorporating ribosomal binding assays for more definitive biological validation. 

## Figures and Tables

**Table 1:** Eight PTC binding compounds structures with their predicted affinity.

Name	Structure	Predicted CSM Affinity (-log10(KD|Ki))	Docking score (ΔG)	Conformation 1 Predicted MM-GBSA Affinity (ΔG)	Conformation 2 Predicted MM-GBSA Affinity (ΔG)
**CEAA**	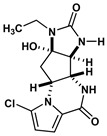	−11.1	−6.8	−50.81	−32.52
**AA**	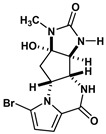	−10.8	−4.59	−48.12	−21.33
**CAA**	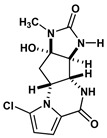	−10.9	−6.65	−32.92	−16.3
**DCEAA**	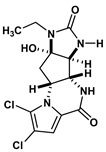	−10.5	−6.13	−15.32	−15.42
**PAA**	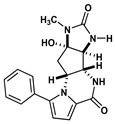	−10.4	−5.62	−25.95	77.85
**EAA**	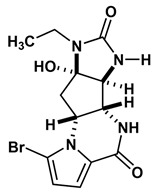	−10.5	−4.24	1293.69	3472.33
**DeBAA**	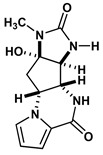	−10.1	−4.16	933.01	49.55
**DeBEAA**	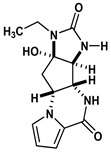	−9.4	−4.38	1279.36	2815.08

## Supplementary Materials

Material and methods on virtual in silico data are available online at https://www.mdpi.com/2218-273X/10/10/1407/s1.
