# Supplementary material for: A Virtual Screening Platform Identifies Chloroethylagelastatin A as a Potential Ribosomal Inhibitor
Source: Biomolecules. 2020 Oct 5;10(10):1407. doi: 10.3390/biom10101407 (PMC7599554; doi:10.3390/biom10101407)
Supplement: Supplementary file 1 [file biomolecules-10-01407-s001.pdf]

SUPPLEMENTARY INFORMATION

A novel virtual screening platform identifies Chloroethylagelastatin A as a potent ribosomal inhibitor

Thomas R. Caulfield[1-4*], Karen E. Hayes[5], Yushi Qiu[1], Matt Coban[1], Joon Seok Oh[6], Amy L. Lane[1,6], Takehiko Yoshimitsu[7], Lori Hazlehurst[8], John A. Copland[1], Han W. Tun[1,9*]

Affiliations

[1]Department of Cancer Biology, Mayo Clinic, Jacksonville, FL; USA
[2] Department of Neuroscience, Mayo Clinic, Jacksonville, FL; USA
[3] Department of Neurosurgery, Mayo Clinic, Jacksonville, FL; USA
[4] Department of Health Sciences Research, Mayo Clinic, Jacksonville, FL; USA
[5] Modulation Therapeutics, Inc., Morgantown, WV; USA
[6] Department of Chemistry, University of North Florida, Jacksonville, FL; USA
[7] Division of Pharmaceutical Sciences, Graduate School of Medicine, Dentistry, and Pharmaceutical Sciences, Okayama University, 1-1-1 Tsushima-naka, Kita-ku, Okayama 700-8530, Japan
[8] Department of Pharmaceutical Sciences, West Virginia University, Morgantown, WV; USA
[9] Department of Hematology/Oncology, Mayo Clinic, Jacksonville, FL; USA

Correspondence:

[*]**Thomas R. Caulfield**, PhD, Department of Neuroscience, Mayo Clinic, 4500 San Pablo Road, Jacksonville, Florida 32224. Phone: +1 904 953 6072; Fax: +1 904 953 6072; E-mail: caulfield.thomas@mayo.edu

[*]**Han W. Tun, MD**, Departments of Hematology/Oncology and Cancer Biology, Mayo Clinic, 4500 San Pablo Road, Jacksonville, Florida 32224. Phone: +1 904 953 2369; Fax +1 904 953 2315; E-mail: tun.han@mayo.edu

SI MATERIAL AND METHODS ON VIRTUAL IN SILICO DATA

Computational docking. Libraries were docked using the Glide algorithm within the Schrödinger software suite[1] using a virtual screening workflow (VSW). The docking was accomplished using a scheme that proceeds from single-precision (SP) through extra-precision (XP) algorithm[2]. These were subjugated to Molecular Mechanics Generalized Born Surface Area (MM-GBSA) solvation free energy calculations to determine ranking profiles. MM-GBSA relies the following functional form:

$$G_s = \frac{1}{8\pi}\left(\frac{1}{\epsilon_0} - \frac{1}{\epsilon}\right)\sum_{i,j}^{N}\frac{q_i q_j}{f_{GB}}, where\ f_{GB} = \sqrt{r_{ij}^2 - a_{ij}^2 e^{-D}}\ and\ D = \left(\frac{r_{ij}}{2a_{ij}}\right)^2, a_{ij} = \sqrt{a_i a_j}$$

with free energy of solvation for solute given by $\Delta G_{solv} = \Sigma_I\ \sigma_I\ ASA_i$, where ASA_i is the accessible surface area of atom i, and σ_i is the solvation parameter of atom i. The top seeded poses were ranked and unfavorable scoring poses were discarded. Each conformer was allowed multiple orientations in the site. Site hydroxyls, such as in serines and threonines, were allowed to move with rotational freedom. Hydrophobic patches were utilized within the VSW as an enhancement. Top favorable scores from initial dockings yielded thousands of poses with the top five poses per compound retained. XP descriptors were used to obtain atomic energy terms like hydrogen bond interaction, electrostatic interaction, hydrophobic enclosure and π-π stacking interaction that result during the docking run. Molecular modeling for importing and refining initial hit compounds and generation of novel small molecules were prepared with LigPrep module[3]. All image rendering used for figures was completed with VMD or Maestro[3].

Novel ligand generation. Using multiple data sources for an accelerated drug discovery process, new compounds were designed using core generation. First, all the cores were separated ("core separation") from each known scaffold, leaving the binding features from the edges of each compound. Potential core fragments were combined from varied sources (core libraries, in-house fragment libraries, and de novo scaffold manipulations), and inserted back into the core slots. Each new core was fused with the existing chemistry on the edges and placed into the appropriate pool. Each pool was filtered using energy minimization, correct bond orders, and ligand preparation with LigPrep and then combined. The entire pool of ligands was expanded to allow for generation of tautomers where appropriate, ionization states over a valid range of pH values, and isomerizations. Reactive functional groups were screened and removed from the dataset. At this point, Z-scoring filtering was applied as a reductive filter for Parkin specificity.

Shape fitting algorithms. Shape similarity models were generated for each compound versus our lead compounds and our initial hit. Upon superposition of the known compound (A) and the de novo compound (B), the following measurements for jointly occupied volume $V_{A\cap B}$ were obtained and normalized by the total volume $V_{A\cup B}$, giving the normalized shape similarity Sim_{AB} ranging from 0 to 1. A pairwise method was employed for faster calculations (600 conformers per second)[4-7]. Using ConfGen each compound was allowed to generate >250 conformers, retaining 20 conformers per rotatable bond and allowing the amide bonds to vary conformation to maximize shape-matching likelihood. Volume was computed for both pharmacophore types and atom types using the Macromodel definition for atom typing. Four alignments per ligand were used, filtering out conformers with similarity below 0.7 and selecting the top shapes according to the retention score. These "stiff" molecules do not require much conformer generation.

Pharmacophore hypothesis generation. Eighteen active compounds that ranged from 20 nM to >3μM and 4 inactives at >10μM as well as 100+ >10μM inactives (decoys) were built into our pharmacophore modeling system. Conformers were generated for all actives, inactives, and test set compounds using ConfGen and a combination of Monte Carlo Multiple Minimum (MCMM) and Mixed torsional/Large-scale low-mode sampling (LC-MOD) within Schrödinger. For ConfGen the number of conformers per rotatable bond was set at 100, maximum number of conformers per structure was set at 1000, sampling was set on "Thorough" mode. Minimization was set to 100 steps preprocess and 50 steps postprocess to eliminate high-energy and/or redundant conformers. The MacroModel options for conformer generation used the OPLS3e force field, GB/SA water solvation treatment and default setting for the maximum relative energy difference and maximum allowed atom deviation.

A common pharmacophore was determined over 4-6 sites and required a match from at least 4 of the 18 actives built into the model. The top variant list included 150 selections based on sites created for all the actives chosen. For pharmacophore generation, we used a tree-based partitioning technique that groups according to inter-site distances (k-points) with a tree depth of five and partition into bins based on a 1.0Å width. Various motifs were scanned to achieve the multiple hypotheses. The initial pharmacophore modeling considered >5,000 hypotheses.

Scoring was based on the identified pharmacophores from each surviving n-dimensional box for the chosen actives and additional information from partial matching of ligand alignments. The quality alignments were determined using three metrics, namely, the alignment score via root-mean-squared-deviation (RMSD), vector score (average cosine of the angles formed by corresponding pairs of vector features acceptor (A), donor (D), aromatic (R)), and volume score (overlap of van der Waals models of non-hydrogen atoms in each pair of structures). Site scores for each alignment were computed to augment the alignment score with a cutoff Calign, which combined the site score, vector score, and volume score with separate weights to yield a combined alignment score for each non-reference pharmacophore that was aligned with reference. All pharmacophores within a box were treated as a reference and the highest one selected as a hypothesis during multi-ligand alignment optimization. The final scoring function (survival score) was: $S = W_{site}S_{site} + W_{vec}S_{vec} + W_{vol}S_{vol} + W_{sel}S_{sel} + W_{rew}^{m} - W_{E}\Delta E + W_{act}A$, where W's represented the weights and S's represented the scores. Actives were scored using vector and site filtering to keep RMSD below 1.200 Å, keep vectors with scores above 0.500, keep the top 30%, keep at least 10 and at most 50 using feature matching tolerances of A 1.00, D 1.00, H 1.50, N 0.75, P 0.75, and R 1.50. Inactives were penalized by adjusting their alignment score, such that, when an inactive matches only k out of n sites, an effective n-point alignment score was computed as follows: $S_{align,n} = \sqrt{W_k S_{align,k}^2 + (1 - W_k)C_{align}^2}$, where W_k=k/n. The final adjusted score was $S_{adjusted} = S_{actives} - W_{inactives}S_{inactives}$. Hypotheses were clustered to tease out pharmacophore model variants with similar scores.

Generation of 3D QSAR models. The 3D QSAR models were built by mapping the chemical features of ligand structures onto a cubic three-dimensional grid space with the smallest grid spacing of 1Å per side. The ligands were first aligned to the set of pharmacophore features for the selected hypothesis utilizing regression of independent variables (structural components) with binary-valued bits in the cubes. The regression $\hat{y} = \mu^{y} + \sum_{i=1}^{m} b_i t_i$ was performed via a partial least squares (PLS) method, where a series of models with increasing number of PLS factors is generated. T-value filter (t-value ≤ 2.0) was used to eliminate independent variables overly sensitive to incremental changes from the training set. For structural components, both atom-based and pharmacophore features were examined. The regression with m PLS factors is given as: , where m = number of PLS factors, b is regression coefficient, vector y represents

activity values in the training set. For the prediction of activities for the new ligands, the following was used: $\hat{y} = \mu^{y} + \sum_{i=1}^{m} (X_{k,i} - \mu_i^{x}) b_i^{x}$, where $k = 1, \ldots, n_T$. Models with high stability were preferred.

SUPPLEMENTARY REFERENCES

1. Mohamadi, F.; Richard, N.G.J.; Guida, W.C.; Liskamp, R.; Lipton, M.; Caufield, C.; Chang, G.; Hendrickson, T.; Still, W.C. Macromodel—an integrated software system for modeling organic and bioorganic molecules using molecular mechanics. *J Comput Chem* **1990**, *11*, 440-467.
2. Friesner, R.A.; Murphy, R.B.; Repasky, M.P.; Frye, L.L.; Greenwood, J.R.; Halgren, T.A.; Sanschagrin, P.C.; Mainz, D.T. Extra precision glide: docking and scoring incorporating a model of hydrophobic enclosure for protein-ligand complexes. *J Med Chem* **2006**, *49*, 6177-6196, doi:10.1021/jm051256o.
3. Maestro-9.4 *Maestro 9.4*, Schrödinger, LLC: New York, NY, 2014.
4. Sastry, G.M.; Dixon, S.L.; Sherman, W. Rapid shape-based ligand alignment and virtual screening method based on atom/feature-pair similarities and volume overlap scoring. *Journal of chemical information and modeling* **2011**, *51*, 2455-2466, doi:10.1021/ci2002704.
5. Sastry, G.M.; Inakollu, V.S.; Sherman, W. Boosting Virtual Screening Enrichments with Data Fusion: Coalescing Hits from Two-Dimensional Fingerprints, Shape, and Docking. *J. Chem. Inf. Model.* **2013**, *53*, 1531-1542.
6. Pala, D.; Beuming, T.; Sherman, W.; Lodola, A.; Rivara, S.; Mor, M. Structure-Based Virtual Screening of MT2 Melatonin Receptor: Influence of Template Choice and Structural Refinement. *J. Chem. Inf. Model.* **2013**, *53*, 821-835.
7. Kalid, O.; Warshaviak, D.T.; Shechter, S.; Sherman, W.; Shacham, S. Consensus Induced Fit Docking (cIFD): methodology, validation, and application to the discovery of novel Crm1 inhibitors. *J. Comput. Aided Mol. Des.* **2012**, *26*, **1217–1228**.
